# A nitrous oxide/oxygen fixed mixture to reduce pain induced by the hypodermic injection: study protocol for a randomized, controlled trial

**DOI:** 10.1186/s13063-024-07919-y

**Published:** 2024-01-13

**Authors:** Jun-Jun Zhang, Ping Yu, Hui Dang, Cheng-Shuang Feng, Xiu-Jiang Long, Wen-Fa Huang, Lei Wang, Jie-Ling Li, Guo-Liang Tian, Jia-Quan Wen, Jun-Hui Mei, Yu-Xiang Li

**Affiliations:** 1 Department of Hematology and Oncology, International Cancer Center, Shenzhen Key Laboratory, Shenzhen University General Hospital, Shenzhen University Clinical Medical Academy, Shenzhen University Health Science Center, Xueyuan Avenue 1098, Shenzhen, 518000 China; 2 Department of Thoracic Surgery, Shenzhen Second People’s Hospital, Sungang West Road 3002, Shenzhen, 518035 China; 3 Department of Radiology, South China Hospital affiliated to Shenzhen University, No.3 Road, Longgang District, Shenzhen, 518100 China; 4 School of Nursing, Ningxia Medical University, 1160 Sheng Li Street, Yinchuan, China

**Keywords:** Randomized controlled trial, Procedural pain, Hypodermic injection, Nitrous oxide, Recombinant human granulocyte colony-stimulating factor

## Abstract

**Background:**

Patients with hematological malignancies received multiple hypodermic injections of recombinant human granulocyte colony-stimulating factor. Procedural pain is one of the most common iatrogenic causes of pain in patients with hematological malignancies. It is also identified as the most commonly occurring problem in clinical care in the Department of Hematology and Oncology at Shenzhen University General Hospital. However, providing immediate relief from pain induced by hypodermic injection of recombinant human granulocyte colony-stimulating factor remains a major challenge. This trial aims to evaluate the safety and analgesic efficacy of a fixed nitrous oxide/oxygen mixture for patients with hematological malignancies and experiencing procedural pain caused by hypodermic injection of recombinant human granulocyte colony-stimulating factor in the department.

**Methods:**

The nitrous oxide/oxygen study is a single-center, randomized, double-blind, placebo-controlled trial involving patients with hematological malignancies who require hypodermic injections of recombinant human granulocyte colony-stimulating factor for treatment. This trial was conducted in the Hematology and Oncology Department of Shenzhen University General Hospital. A total of 54 eligible patients were randomly allocated to either the fixed nitrous oxide/oxygen mixture group (*n* = 36) or the oxygen group (*n* = 18). Neither the investigators nor the patients known about the randomization list and the nature of the gas mixture in each cylinder. Outcomes were monitored at the baseline (T0), immediately after hypodermic injection of recombinant human granulocyte colony-stimulating factor (T1), and 5 min after hypodermic injection of recombinant human granulocyte colony-stimulating factor (T2) for each group. The primary outcome measure was the score in the numerical rating scale corresponding to the highest level of pain experienced during hypodermic injection of recombinant human granulocyte colony-stimulating factor. Secondary outcomes included the fear of pain, anxiety score, four physiological parameters, adverse effects, total time of gas administration, satisfaction from both patients and nurses, and the acceptance of the patients.

**Discussion:**

This study focused on the safety and analgesic efficacy during hypodermic injection of recombinant human granulocyte colony-stimulating factor procedure. Data on the feasibility and safety of nitrous oxide/oxygen therapy was provided if proven beneficial to patients with hematological malignancies during hypodermic injection of recombinant human granulocyte colony-stimulating factor and widely administered to patients with procedural pain in the department.

**Trial registration:**

Chinese Clinical Trial Register, ChiCTR2200061507. Registered on June 27, 2022. http://www.chictr.org.cn/edit.aspx?pid=170573&htm=4

**Supplementary Information:**

The online version contains supplementary material available at 10.1186/s13063-024-07919-y.

## Introduction

### Background and rationale

Hematological malignancies (HMs) are heterogeneous neoplasms that affect lymphoid, myeloid, and stem cells exhibiting high malignancy and differentiation disorder. Clinical manifestations of HMs typically include anemia, thrombocytopenia, leukopenia and varying degrees of an immunocompromised state. Owing to their high recurrence rate and poor prognosis, HMs are considered clinically incurable. HMs are among the top 10 diseases with the highest incidence and mortality globally [1]. The American Cancer Society reports that the USA has 89010 new HM cases, comprising 4.6% of new cancer cases in the same period. About 14020 patients have died from HM, constituting approximately 2.3% of cancer deaths [2]. The incidence of HM is 63.2 per 100,000 individuals, according to the UK’s population-based Hematological Malignancy Research Network [3]. In China, the morbidity and mortality of HM are also high. A total of 4,568,754 new cancer cases and 3,002,899 cancer deaths have been reported; 8.6% of the cases are associated with leukemia, and 9.0% are attributed to lymphoma [4]. Mortality attributed to leukemia comprises 5.6% of all cancer deaths nationwide, and that attributed to lymphoma is 5.2% [4, 5]. Overall morbidity and mortality have exhibited an increasing trend in recent years. Thus, HMs have presented a significant challenge in public health worldwide.

Differences in the type of HM indicate variations in chemotherapy regimens in clinical practice. They also differ in that chemotherapy drugs are selected, the density and intensity of drug doses are determined, and sequential therapy or combination therapy is used for chemotherapy regimens. However, these differences are significantly associated with the risk of febrile neutropenia (FN). Predictably, the occurrence of FN can lead to adverse consequences, including the dosage reduction of chemotherapy drugs and the delay or even cancelation of the chemotherapy cycle. Consequently, the clinically expected goals of patients receiving chemotherapy may be largely impeded. The most serious outcome is poor control of a malignancy that exhibits good response and is potentially curable, which can be life-threatening to the patients and even leads to death [6, 7]. Similar results have been observed with radiation therapy. Patients tend to suffer from hematopoietic acute radiation syndrome characterized by neutropenia and thrombocytopenia, which can lead to death due to infection or bleeding. Therefore, active prevention or treatment of FN is crucial to ensure the successful completion of dose-dense chemotherapy or full-dose chemotherapy plans for the patients. Recombinant human granulocyte colony-stimulating factors (G-CSF) are key cytokines that can regulate neutrophil production and differentiation as well as act on neutrophil surface receptors at different developmental stages. They play an active role in the prevention or treatment of chemotherapy-induced myelosuppression and FN in HM patients [6–8].

However, multiple hypodermic injections (Hs) of recombinant human granulocyte colony-stimulating factor (rhG-CSF) are necessary for HM patients during the chemotherapy cycle. The rhG-CSF is used for H at a vertical distance of just a few millimeters from the layer of dermal tissue rich in pain-sensing nerve, allowing H to stimulate the nerves [9]. Therefore, HM patients are expected to experience sharp procedural pain induced by H. In addition, rhG-CSF is a glycoprotein with a relatively large molecular weight (containing 174 amino acids and with a molecular weight of about 20,000), which further stimulates the sensory nerves of the skin tissue. Patients administered with H of rhG-CSF thus feel excruciating pain [6, 8]. In addition, several and repeated painful stimuli lead to increased sensitivity and decreased tolerance to pain in HM patients. Consequently, some painless operations can also cause pain in HM.

Repeated Hs of rhG-CSF aggravate procedural pain and heighten the fear of Hs in rhG-CSF. Results related to the study [10] suggest that fear of pain can enhance the pain experience of patients to a certain extent. As an important factor affecting pain, fear of pain may trigger a series of fear-related activities or behaviors. Therefore, the method by which pain induced by H of rhG-CSF in patients with HM is alleviated and reduced has become an important clinical topic in the hematology and oncology department.

Dilute nitrous oxide is a premixture consisting of 65% nitrous oxide (N_2_O) and 35% oxygen (O_2_) [11, 12]. A mixture of this concentration can rapidly produce an analgesic effect [13]. Its analgesic principle is that the mixed gas enters the lung via the respiratory tract and then goes into the pulmonary circulation by diffusion. The mixture is then stabilized in the blood [11–15]. Finally, it acts as an analgesic role by stimulating the endorphin system and antagonizing the N-methyl-D-aspartic acid receptor while retaining consciousness of behavioral management technology [11]. It is characterized by the following: the ability to easily cross the blood–brain barrier to the brain; rapid onset (about 30 s); short duration of action (2–3 min); the least toxicity; no stimulation to the respiratory tract; low blood/gas solubility ratio; and no damage to the heart, lung, liver, and kidneys [11–15]. Its most essential features are safety, effectiveness, and noninvasiveness. This gas concentration is widely used for traumatic pain in the emergency department, postherpetic neuralgia, lumbar puncture-induced pain, breakthrough pain, and postoperative dressing change for perianal abscess [11–15]. It has also formed an expert consensus on the analgesic application of dressing change in burns [16]. The current study intends to apply this concentration-diluted N_2_O/O_2_ quick-acting analgesic technique to control procedural pain in patients with HM.

### Objectives

This trial aims to evaluate the safety and analgesic efficacy of a fixed mixture of N_2_O/O_2_ for patients with HMs, with procedural pain induced by H of rhG-CSF, in the hematology and oncology department. We hypothesize that the fixed N_2_O/O_2_ mixture can effectively relieve procedural pain and reduce patient anxiety caused by it.

### Trial design

**Fig. 1:**
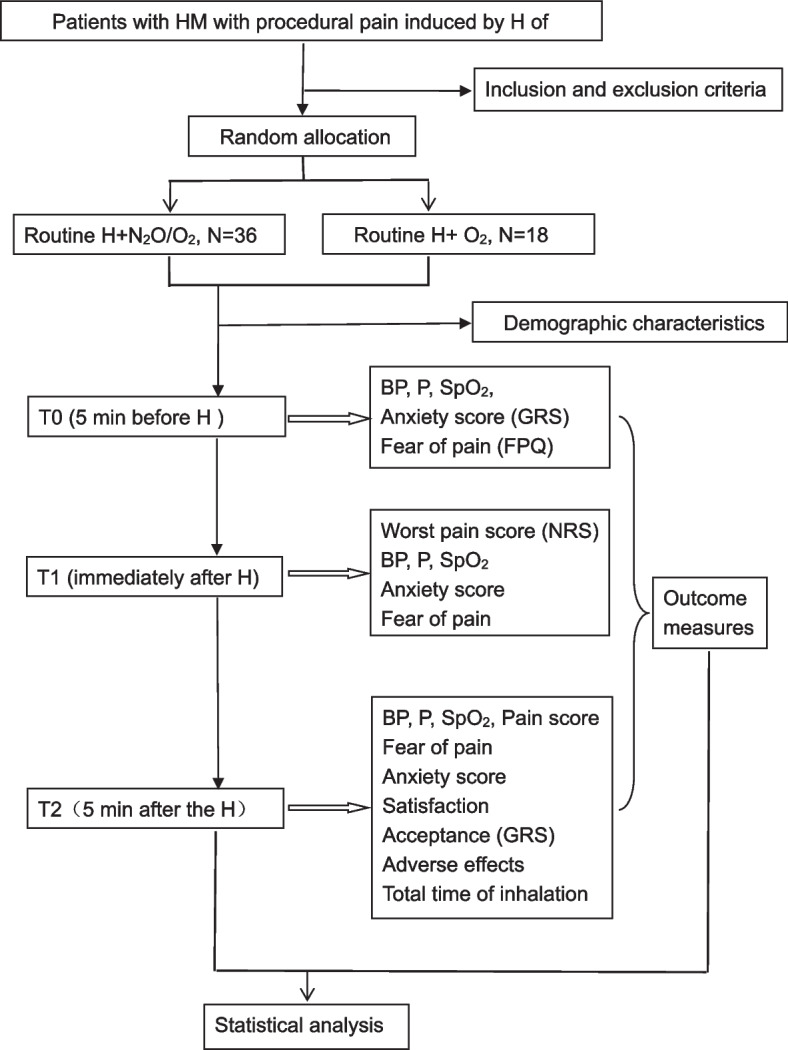
Study design framework. BP, blood pressure; FPQ, fear of pain questionnaire; GRS, graphic rating scale; H, hypodermic injection; N_2_O, nitrous oxide; NRS, numerical rating scale; O_2_, oxygen; P, pulse; rhG-CSF, recombinant human granulocyte colony-stimulating factor; SpO2, oxygen saturation

The N_2_O/O_2_ study was a single-center, randomized, double-blind, placebo-controlled trial, which is subjected to a superiority test. The trial protocol was drafted according to the guidelines of the Standard Protocol Items Recommendations for Interventional Trials (Additional file 1). It was a schedule of enrollment, interventions, and assessments summarized in Table 1. A flow diagram of the entire study was shown in Fig. 1. The study protocol was approved by the Ethics Committee of Shenzhen University School of Medicine (M202200412).

**Table 1:** Schedule of enrolment, interventions, and assessments

Time points	Study period					
	Enrolment	Allocation	T0	H of rhG-CSF procedures	T1	T2
Enrolment						
Eligibility screen	√					
Informed consent	√					
Allocation		√				
Demographic characteristics			√			
Baseline data			√			
Interventions				√		
N_2_O/O_2_ group				√		
O_2_ group						
Assessments			√		√	√
BP, P, SpO_2_			√		√	√
Pain score			√		√	√
Fear of pain					√	√
Anxiety score						√
Satisfaction						√
Acceptance (GRS)						√
Adverse effects						√
Total time of inhalation						

*GRS* graphic rating scale, *H* hypodermic injection, *rhG-CSF* recombinant human granulocyte colony-stimulating factor

## Methods

### Study setting

This study was conducted in the hematology and oncology department of SUGH; the department is the key discipline of SUGH and the Clinical Division of International Cancer Center. It is a comprehensive blood disease treatment center integrating basic translational medicine and clinical diagnosis and treatment services. About 2000 patients with HMs undergo treatment in this center annually. Thus, a sufficient number of patients to obtain a suitable sample size. We planned to complete recruitment within 8 months.

### Eligibility criteria

All HM patients with procedural pain induced by H of rhG-CSF was invited to participate in the trial. We recruited patients in accordance with the following inclusion criteria:

1. Patient met the clinical diagnostic criteria for HM and is receiving H of rhG-CSF® (0.5 mL:150 μg and 1 mL:300 μg) [17]
2. Experiencing procedural pain from H of rhG-CSF (pain score ≥ 4 in accordance with the numerical rating scale [NRS]) [12]
3. No signs of pain before H
4. Voluntarily participates and signs informed consent
5. Unable to use the self-managed device

Exclusion criteria include one of the following conditions:

1. Contraindications for N_2_O/O_2_ inhalation (hemodynamic instability, vitamin B_12_ deficiency, intracranial hypertension, pneumothorax, intestinal obstruction, epilepsy, pulmonary embolism or facial fractures, gas embolism, severe drug dependence and mental disorders, pregnancy, bowel obstruction, Caisson’s disease [16])
2. Life-threatening situations or instability of vital signs
3. Difficulty reporting pain

Enrolled patients with the following conditions during the trial was considered dropouts:

1. Unable to follow the test procedure (severe adverse effects occur or patient withdraws from the trial voluntarily)
2. Poor compliance

All participating nurses had worked in the department for > 3 years and mastered multiple H methods. The nurses were willing to join and accept training from design proposal sponsors. Finally, 4 nurses who met all aforementioned criteria was enrolled in this trial.

### Who will take informed consent?

For patients who wish to participate, their medical records were reviewed to select patients who met the criteria for inclusion and exclusion. An explanation for their exclusion was given to patients who cannot be enrolled in this study. We explained the trial in detail during the process, including the purpose, methods, benefits, potential risks, and patient rights, among others. Written informed consent bearing the signature of the patient was obtained from each patient. The consent indicated that the participation of each in the study was voluntary and that publicizing research findings was permitted, including the collection and use of patient data and biological specimens, pictures, and videos.

### Additional consent provisions for collection and use of participant data and biological specimens

It is in the informed consent form.

## Interventions

### Explanation for the choice of comparators

All HM patients under treatment and receiving the H of rhG-CSF were assessed for inclusion/exclusion by our research team in the department. After providing their informed consent, eligible patients were randomly assigned to either a control group or an intervention group. During the intervention, patients received the N_2_O/O_2_ mixture to control the procedural pain induced by H of rhG-CSF in the intervention, compared with O_2_ in the control group [12]. Patients could inhale gas via a specially designed facemask, which is a one-way valve [13]. In the current study, gas inhalation will continue for no more than 15 min, as required by the study design [18]. To eliminate variations in injection methods and operators as a factor, we arranged for a designated nurse to administer the H of rhG-CSF at a constant speed.

### Intervention description

The project manager of the trial escorted the patients who had been grouped to a dedicated injection room, where the trial was conducted. Before conducting the trial, the researchers provided patients with guidance on how to prepare, including proper positioning and inhalation techniques, and how to use NRS to report pain intensity, among others. Data collectors reiterated that patients have the right to stop or withdraw from the trial at any time if they felt unwell during the study. The demographic and baseline data of the subjects then was collected by the data collector 5 min before the intervention (T0). Timing of the trial will begin as the researchers help the patient put on a mask and inhale. After 15 s, the operating nurse administered the H of rhG-CSF for about 20-40 s. Various physiological indicators of patients were collected by the data collector at T1 (immediately after H). Once the administration of the H of rhG-CSF was completed, the researcher took the mask off the patient and closed the air valve. The physiological indicators of the patients were recorded again 5 min after the intervention (T2). The data researcher assessed the level of fear and anxiety associated with procedural pain for each patient. The patients were observed and asked to report adverse reactions related to gas inhalation throughout the trial. In addition, patients were asked about their level of satisfaction with the pain relief provided and their acceptance of the analgesic method. Finally, patients were returned to the ward.

### Criteria for discontinuing or modifying allocated interventions

The following adverse reactions [16] may occur: (1) occasional nausea and vomiting with excessive sedation; (2) hypotension, bradycardia, and other arrhythmias in several patients; (3) headaches and other kinds of discomfort in a small number of patients. If any unexpected adverse reaction occurs, the study would be terminated immediately, and the patient be given oxygen to inhale.

### Strategies to improve adherence to interventions

Strategies to improve participant retention had to be discussed by our team in advance. Thus, the following strategies had been developed [12, 18]: (1) researchers elaborated on the intervention and data collection of this study; (2) the entire intervention process was simulated for the patient to know, and possible adverse reactions and management measures were also be presented; (3) the physiological parameters of the patients during the trial were closely monitored to ensure safety. Patients had the right to withdraw from the study at any time without affecting subsequent treatment. Data from patients who dropped out of the study was recorded, and the reasons for the withdrawal were analyzed. Researchers also recorded patient adherence to the trial.

### Relevant concomitant care permitted or prohibited during the trial

Patients enrolled in this study could not receive interventions from other trials during the implementation of the intervention.

### Provisions for post-trial care

All study subjects were HM patients who had low immunity and thus were closely monitored as part of the standard of care before, during, and after study participation. Whether or not they participated in the study did not affect their follow-up treatment and care.

## Outcome measures and data collection

### Participant timeline

The demographic details (age, sex, nationality, weight, and height) and clinical characteristics (including relevant medical history, HM classification, physiological parameters, fear of pain) of the patients were collected at T0 in a standardized case report form (CRF). Blood pressure, pulse, and oxygen saturation were included in the physiological parameters.

### Primary outcome measure

In this study, the primary outcome measure was the score corresponding to the worst pain experienced during the H of rhG-CSF. The pain score was measured by the data collector via NRS at T1 and T2. The patients chose a number on the NRS to indicate their pain level based on their own pain experience. The NRS ranges from no pain (0) at all to the most intense pain (10) possible; 1 to 9 means that the degree of pain gradually increases.

### Secondary outcome measures

Secondary outcomes included the following:

1. Fear of pain. It was assessed using the Fear of Pain Questionnaire (FPQ) at T0, T1, and T2. The patients self-rated their fear of pain based on the operating pain caused by the H of rhG-CSF. The FPQ is rated on a scale of 0 to 5. A higher FPQ score indicates greater fear of pain [10].
2. Anxiety score. It was collected using a graphic rating scale (GRS) ranging from none (0) to extreme (10) at T0, T1, and T2 [19].
3. Physiological parameters. These measures were monitored using an electronic manometer (OMRON, HEM-7120) and a pulse oximeter (PC-60B) at T0, T1, and T2.
4. Satisfaction with pain relief as perceived by both the patients and the nurses. The data collector determined the degree of satisfaction at T2 on a five-point satisfaction scale (1 = very dissatisfied; 5 = very satisfied).
5. Patient acceptance of the analgesic method. The level of acceptance at T2 would be determined by answering the question “How accepting are you with the overall pain management during the H of rhG-CSF?” The score ranges from non-acceptance (“0”) to complete acceptance (“10”) with GRS.
6. Adverse effects. Any observed adverse effect associated with inhaling gas should be accurately assessed and documented in the CRF during the intervention. The N_2_O/O_2_ mixture is characterized by few adverse reactions that can be relieved within a few minutes after cessation of inhalation and eventually fully recover. Any adverse reactions were fully reversible within 5 min. Total inhalation time also was registered at the CRF.

### Sample size

Sample size was determined based on our previous studies on traumatic pain in the emergency department [11]. In the current study, pain severity as the primary outcome measure was used to calculate the sample size. The NRS scores after interventions were 3.0 ± 1.9 in the N_2_O/O_2_ mixture group and 6.3 ± 2.2 in the O_2_ group. On the basis of the result, the N_2_O/O_2_ mixture may decrease the NRS scores by 6.95 points. The following formula is used to calculate the sample size of this study.

$${\textrm{n}}_1={\textrm{n}}_2=2{\left[\frac{\left({\mu}_a+{\mu}_{\beta}\right)}{\delta /\sigma}\right]}^2+\frac{1}{4}{\mu}_{\alpha}^2$$

We set *α* (type I error) to 0.05 (5%) for the two-tailed test with *β* = 90% (0.10) type II error rate of the measure. Thus, *δ* was **|**μ_1_ − μ_2_**|** = 3.3. The overall standard deviation (*σ*) between the two groups was about 2.73 (*n* = 60). Therefore, the estimated required sample size of each group was 15. A 20% drop-out rate was required for the study. To reduce the underpower, we planned to recruit 54 patients between the N_2_O/O_2_ group and the O_2_ group in a 2:1 ratio. In practice, 36 patients were randomly assigned to the intervention group, as opposed to the 18 patients in the control group.

### Recruitment strategy and approach for consent

Patients in this study were recruited at the department. We published our recruitment information on the WeChat public platform of the department from December 1, 2022, to August 1, 2023. Referrals from medical staff were also an important recruitment avenue. In addition, we put up recruitment posters in highly visible places, such as outpatient and inpatient departments of hospitals.

### Randomization, allocation concealment

In this study, a randomized list was computer-generated and then used by an independent statistician who participated in the trial. Eligible patients were randomly allocated into the N_2_O/O_2_ or O_2_ group in a 2:1 ratio. The list was placed in a sealed and opaque envelope, which then is kept in a special office to maintain confidentiality. The randomization was not divulged to the researcher or patients to create a double-blind environment. However, this information was accessible to the manager in charge of gas distribution for consultation purposes, preventing any potential confusion on the part of the manager.

## Assignment of interventions: blinding

### Who will be blinded

The manager was not involved in the intervention or data collection of the study. In addition, the manager did not communicate with members of the research team about gas contents and distribution. The N_2_O/O_2_ mixture and O_2_ were supplied by the Ningfeng Oxygen Company. Two gasses were packaged in identical gas cylinders and could only be distinguished using a label (A or B). In this trial intervention, patients, investigators, and data collectors did not know what was in gas cylinders or what the letters A and B stood for. Following the double-blind requirement, the project manager continued blinding until all tests are completed.

### Procedure for unblinding if needed

Only the project manager who was responsible for gas distribution can consult this information.

## Data management

### Plans for assessment and collection of outcomes

See the “Outcome measures and data collection” section for details.

### Plans to promote participant retention and complete follow-up

It was described in the “Strategies to improve adherence to interventions” section in detail.

### Data storage and confidentiality

All team members participating in the study received training on the collection, management, storage, and confidentiality of study data before the study was conducted. Data was stored in paper and a computer database form. And data confidentiality was maintained, based on national data and privacy legislation. All coded paper data was stored in a locked filing cabinet dedicated to trial. A Microsoft Access database (Microsoft Office 2010, USA) was used to accomplish all data inputs in the present study [18]. The data was entered electronically into the database (password-protected) by two trained researchers responsible for data entry by using the double-entry method [18, 19]. Then, they individually checked the electronic database for accuracy. To prevent data loss, we performed incremental backups daily. In accordance with the design of the study, the biological samples included five vital signs of the patient, which were retained as required. All data were saved in a research office located in the department of SUGH.

Throughout the trial, information about any potential and registered patients were kept confidential and accessible only to primary investigators [12]. Anonymized patients were assigned a unique numeric code (ID number) instead of names [19]. For all data collected, management, and confidentiality, the data safety and monitoring committee (DSMC) monitored the database periodically to promote data quality. Researchers will have access to the statistical analysis of the data after the experiment is complete.

### Plans for collection and storage of biological specimens for analysis in this trial use

These data and biology are provided for manuscript publication only. After reaching the specified storage time, they will be destroyed.

### Statistical analysis

In this study, all statistical analyses will be processed in accordance with the intention-to-treat principle [15]. In addition, the statistician who performed the randomization of patients and being independent of the trial had established a complete statistical analysis plan. All patients subject to randomization should be included in the analysis. Missing values from all patients randomized will be handled using multiple imputations or the last-observation-carried-forward method, as appropriate [15, 18]. Data analysis will be performed using SPSS version 22.0 (Chicago, IL, USA). *P* values (two-sided) < 0.05 will be considered statistically significant.

Demographic details and clinical characteristics will be presented as mean ± SD (data with normal distribution) or median and interquartile range (data with non-normal distribution) if applicable. The Kolmogorov-Smirnov test will be used to determine normality. The primary outcome measure between the two groups (“N_2_O/O_2_” and “O_2_”) will be assessed using Student’s *t* test. It will also be considered to determine the fear of pain and anxiety score between the two groups. The difference in satisfaction from pain relief as perceived by patients and nurses will be tested using the Kruskal-Wallis test. However, patient acceptance of the analgesic method will be analyzed using the chi-squared test or Fisher’s exact test. The chi-squared test will also be used to compare the difference in adverse effects. Physiological parameters are assessed using the repeated measures analysis of variance.

Considering the nature and duration of the intervention, we thought that an interim analysis was not performed. However, the subgroup analysis will be stratified, based on the dose of the H of rhG-CSF, to explore whether significant differences in doses exist.

### Data monitoring

The DSMC was established to oversee the safety of the trial patients in accordance with the Guideline on Clinical Trial Data Monitoring Committees. It consisted of a chairperson (a senior statistical specialist) and four members (two senior nurses, a pain management specialist, and a member of the ethics committee of the SUGH). The temporary organization was responsible for advocating the interests of patients, evaluating the safety and efficacy of study procedures, and overseeing the overall conduct of the study [20]. The DSMC members reviewed the entire data processing monthly to ensure the standardization of research, the accuracy and integrity of data, and the reliability and credibility of the results [20]. Modifications (major or minor) to the protocol was discussed at the DSMC meeting. If necessary, a modification plan was proposed by the DSMC to the ethics committee [12]. Information on clinical trial registration was also updated. Other project issues (e.g., deviations, patient withdrawal, and from the trial) were also discussed and solved at the meeting. No audit was planned.

### Dissemination plan

We intend to publish all research results (containing negative or positive results) in a peer-reviewed journal for the end of the study. However, the personal information of the patients will not be disclosed. We will follow the guidelines recommended by the International Committee of Medical Journal Editors to resolve authorship for a trial publication.

## Discussion

Patients with HM frequently suffer from FN due to radiotherapy and chemotherapy, particularly after intensive bone marrow deprivation chemotherapy. Using the rhG-CSF can not only prevent or reduce the occurrence of agranulocytosis and shorten the duration; it also accelerates the recovery of granulocyte count, thereby decreasing the risk of infectious fever [6–8]. Patients with HM experience multiple Hs of rhG-CSF. Procedural pain is one of the main iatrogenic causes of pain in HM patients and is the most common problem in clinical care in the Department of Hematology and Oncology. It can lead to anxiety, fear, and other psychological conditions in patients, which subsequently aggravates their sense of pain. HM patients ultimately fall into a vicious cycle of “pain–tension–more pain ” [9, 10].

Analgesia via diluted nitrous oxide inhalation has been used clinically for more than a century and remains widely used in clinical practice locally and globally [11–15]. Our previous studies have validated the efficacy and safety of appropriate concentrations of diluted nitrous oxide for postherpetic neuralgia, lumbar puncture-induced pain, breakthrough pain, and traumatic pain [12–15]. The N_2_O/O_2_ used in this study is a mixed gas formed by dissolving oxygen in liquid nitrous oxide and vaporizing it, with the volume fraction of N_2_O set to 65%.

This study was thus far the first randomized controlled trial in the hematology and oncology department for evaluating the safety and analgesic efficacy of the 65% N_2_O/O_2_ mixture in HM patients with Hs of rhG-CSF in China. Patients with HM will benefit if the 65% N_2_O/O_2_ mixture is proved to be beneficial during H of rhG-CSF. It can then be used for the rapid relief of severe acute pain in the department.

## Limitations

The trial had several limitations. In accordance with the study design, we did not record the pain duration (in hours) after the intervention to assess the long-term effects of analgesia. Multi-center clinical research is needed to increase the power of the trial in multiple areas.

## Trial status

We planned that recruitment of patients for the trial commenced in 1 December 2022. Patient recruitment had come to an end on 1 August 2023. The whole trial is expected to be completed by 2025.

### Supplementary Information

**Additional file 1.** SPIRIT 2013 Checklist.

**Additional file 2.** Informed Consent Form.

## Data Availability

To date, there are no baseline or pilot data in this protocol. At the end of this study, the final trial data set and statistical code will be obtained from the corresponding author with the reasonable request.

## Abbreviations

ACS: American Cancer Society
CRF: Case report form
DSMC: Data safety and monitoring committee
FN: Febrile neutropenia
FPQ: Fear of Pain Questionnaire
GRS: Graphic rating scale
Hs: Hypodermic injections
H-ARS: Hematopoietic acute radiation syndrome
HMs: Hematological malignancies
N_2_O: Nitrous oxide
NRS: Numerical rating scale
O_2_: Oxygen
rhG-CSF: Recombinant human granulocyte colony-stimulating factor
SPIRIT: Standard Protocol Items Recommendations for Interventional Trials
