# Supplementary material for: A nitrous oxide/oxygen fixed mixture to reduce pain induced by the hypodermic injection: study protocol for a randomized, controlled trial
Source: Trials. 2024 Jan 13;25:47. doi: 10.1186/s13063-024-07919-y (PMC10790270; doi:10.1186/s13063-024-07919-y)
Supplement: Supplementary file 2 — Additional file 2. Informed Consent Form. [file 13063_2024_7919_MOESM2_ESM.docx]

**Informed Consent Form**

**Project name:** A nitrous oxide/oxygen fixed mixture to reduce pain induced by the hypodermic injection: study protocol for a randomized, controlled trial

**Version:** 1.0

**Research unit**: Shenzhen University General Hospital

**Principal investigator**: Jun-Hui Mei, Jun-Jun Zhang

You are being invited to participate in a clinical research study. This informed consent form gives you some information to help you decide whether to participate in this clinical study. Please read it carefully and ask the investigator in charge of the study if you have any questions.

Your participation in this study is voluntary. This study has been reviewed by our Institutional Ethics Review Board.

If you have any questions or concerns about this study, you may contact: Jun-Hui Mei ,Jun-Jun Zhang at 0755-21839999 or 15909584020.

A total of 54 subjects were planned to be included in this study.

**1.Aim and objective**

This trial aims to evaluate the safety and analgesic efficacy of a fixed mixture of N_2_O/O_2_ (N_2_O: Nitrous Oxide; O_2_: Oxygen) for patients with HMs, with procedural pain induced by H (H: Hypodermic Injection) of rhG-CSF (rhG- CSF: Recombinant Human Granulocyte Colony-stimulating Factor), in the hematology and oncology department. We hypothesize that the fixed N_2_O/O_2_ mixture can effectively relieve the procedural pain and reduce patient anxiety caused by it.

**2.Background**

Hematological malignancies (HMs) are heterogeneous neoplasms that affect lymphoid, myeloid, and stem cells exhibiting high malignancy and differentiation disorder. Clinical manifestations of HMs typically include anemia, thrombocytopenia, leukopenia and varying degrees of being immunocompromised state. Differences in the type of HM indicate variations in chemotherapy regimens in clinical practice. They also differ in that chemotherapy drugs are selected, the density and intensity of drug doses are determined, and sequential therapy or combination therapy is used for chemotherapy regimens. However, these differences are significantly associated with the risk of febrile neutropenia (FN).

Recombinant human granulocyte colony- stimulating factor (G-CSF) are key cytokines that can regulate neutrophil production and differentiation, as well as act on neutrophil surface receptors at different developmental stages. They play an active role in the prevention or treatment of chemotherapy-induced myelosuppression and FN in HM patients. However, multiple hypodermic injections (Hs) of recombinant human granulocyte colony-stimulating factor (rhG-CSF) are necessary for HM patients during the chemotherapy cycle. The rhG-CSF is used for H at a vertical distance of just a few millimeters from the layer of dermal tissue rich in pain-sensing nerve, allowing H to stimulate the nerves. Therefore, HM patients are expected to experience sharp procedural pain induced by H. In addition, rhG-CSF is a glycoprotein with a relatively large molecular weight (containing 174 amino acids and with a molecular weight of about 20000), which further stimulates the sensory nerves of the skin tissue. Patients administered with H of rhG-CSF thus will feel excruciating pain. In addition, several and repeated painful stimuli lead to increased sensitivity and decreased tolerance to pain in HM patients. Consequently, some painless operations can also cause pain in HM.

Dilute nitrous oxide is a premixture consisting of 65% nitrous oxide (N_2_O) and 35% oxygen (O_2_). A mixture of this concentration can rapidly produce an analgesic effect. Its analgesic principle is that the mixed gas enters the lung via the respiratory tract and then goes into the pulmonary circulation by diffusion. It is characterized by the following: ability to easily cross the blood–brain barrier to the brain; rapid onset (about 30 s); short duration of action (2-3 min); the least toxicity; no stimulation to the respiratory tract; low blood/gas solubility ratio; and no damage to the heart, lung, liver, and kidneys. Its most essential features are safety, effectiveness, and noninvasiveness.

The current study intends to apply this concentration-diluted N_2_O/O_2_ quick-acting analgesic technique to control procedural pain in patients with HM.

**3. Inclusion and exclusion criteria:**

You may participate in this study if you meet the following requirements.

Inclusion criteria include: (1) Patient meets the clinical diagnostic criteria for HM and is receiving H of rhG-CSF® (0.5 mL:150 μg and 1 mL:300 μg). (2) Experiencing procedural pain from H of rhG-CSF (pain score≥4 in accordance with the Numerical Rating Scale [NRS]). (3) No signs of pain before H.

1. Voluntarily participates and signs informed consent. (5) Unable to use the self-managed device.

Exclusion criteria include one of the following conditions: (1) Contraindications for N_2_O/O_2_ inhalation (hemodynamic instability, vitamin B12 deficiency, intracranial hypertension, pneumothorax, intestinal obstruction, epilepsy, pulmonary embolism or facial fractures, gas embolism, severe drug dependence and mental disorders, pregnancy, bowel obstruction, Caisson’s disease). (2) Life- threatening situations or instability of vital signs. (3) Difficulty reporting pain.

Enrolled patients with the following conditions during the trial will be considered dropouts:

(1)Unable to follow the test procedure (severe adverse effects occur or patient withdraws from the trial voluntarily). (2)Poor compliance.

**4. Research process :**

All HM patients under treatment and receiving the H of rhG-CSF will be assessed for inclusion/exclusion by our research team in the department. After providing their informed consent, eligible patients will be randomly assigned to either a control group or an intervention group. During the intervention, patients will receive the N_2_O/O_2_ mixture to control the procedural pain induced by H of rhG-CSF in the intervention, compared with O_2_ in the control group. Patients can inhale gas via a specially designed facemask, which is a one-way valve. In the current study, gas inhalation will continue for no more than 15 min, as required by the study design. To eliminate variations in injection methods and operators as a factor, we require a designated nurse to administer the H of rhG-CSF at a constant speed. The project manager will escort the patients who have been grouped to a dedicated injection room, where the trial will be conducted. Before conducting the trial, the researchers will advise patients to be well prepared, orienting them with regard to the correct position, the proper way to inhale, and the use of NRS to report pain intensity, among others. Data collectors will reiterate that patients have the right to stop or withdraw from the trial at any time if they feel unwell during the study. The demographic and baseline data of the subjects will then be collected by the data collector 5 min before the intervention (T0). Timing of the trial will begin as the researchers help the patient put on a mask and inhale. After 15 s, the operating nurse will administer the H of rhG-CSF for about 20-40 s. Various physiological indicators of patients will be collected by the data collector at T1 (immediately after H). Once the administration of the H of rhG-CSF is completed, the researcher will take the mask off the patient mask and close the air valve. The physiological indicators of the patients will be recorded again 5 min after the intervention (T2). The data researcher will assess the level of fear and anxiety associated with procedural pain for each patient. The patients will be observed and asked to report adverse reactions related to gas inhalation throughout the trial. In addition, the patients will be asked about their level of satisfaction with the pain relief provided and their acceptance of the analgesic method. Finally, the patient will be returned to the ward.

1. **Collection and use of participant data and biological specimens**

**(1). Collection of participant data and biological specimens**

The demographic details (age, sex, nationality, weight, and height) and clinical characteristics (relevant medical history, HM classification, physiological parameters, fear of pain) of the patients will be collected at T0 in a standardized case report form (CRF). Blood pressure, pulse, and oxygen saturation will be included in the physiological parameters. In this study, the primary outcome measure is the score corresponding to the worst pain experienced during the H of rhG-CSF. The pain score will be measured by the data collector via NRS at T1 and T2. The patients will choose a number on the NRS to indicate their pain level based on their own pain experience. The NRS ranges from no pain (“0”) at all to the most intense pain (“10”) possible; “1”to “9” means that the pain degree gradually increases.

Secondary outcomes will include the following:

Fear of pain. It will be assessed using the Fear of Pain Questionnaire (FPQ) at T0, T1, and T2. The patients will self-rate their fear of pain on the basis of the operating pain caused by the H of rhG-CSF. The FPQ is rated on a scale of 0 to 5. A higher FPQ score indicates greater fear of pain [10].

Anxiety score. It will be collected using a graphic rating scale (GRS) ranging from none (“0”) to extreme (“10”) at T0, T1, and T2.

Physiological parameters. These measures will be monitored using an electronic manometer (OMRON, HEM-7120) and a pulse oximeter (PC-60B) at T0, T1, and T2.

Satisfaction with pain relief as perceived by both the patients and the nurses. The data collector will determine the degree of satisfaction at T2 on a five-point satisfaction scale (1=very dissatisfied; 5=very satisfied).

Patient acceptance of the analgesic method. The level of acceptance at T2 will be determined by answering the question “How accepting are you with the overall pain management during the H of rhG-CSF?” The score ranges from non-acceptance (“0”) to complete acceptance (“10”) with GRS.

Adverse effects. Any observed adverse effect associated with inhaling gas should be accurately assessed and documented in the CRF during the intervention.

(2). **Use of participant data and biological specimens**

These data and biological specimens are for publication purposes only. And they will be destroyed after the required storage time is reached.

**6. Risks and discomforts:**

The N_2_O/O_2_ mixture is characterized by few adverse reactions that can be relieved within a few minutes after cessation of inhalation, and eventually full recovery.

**7. Possible benefits:**

The cost of related to the study is covered by the research grant.

**8. Possible additional costs in this study?**

There are no additional expenses for you to participate in this research project.

**9. As a research subject, you have the following responsibilities:**

Provide truthful information about your medical history and current medical condition; tell the study doctor about any discomfort you experience during this study; refrain from taking restricted medications, foods, etc.; and tell the study doctor if you have participated in other studies recently or are currently participating in other studies.

**10. Privacy and confidentiality:**

If you decide to participate in this study, we will make every effort to protect your personal privacy to the extent permitted by law. Any public reporting of the results of this study will not disclose any personal information about you. The physician in charge of the study and other researchers will use your medical information to conduct the study. This information may include your name, address, telephone number, medical history, and information obtained at the time of your study visit. Information that identifies you will not be released to members outside the study team unless your permission is obtained. All study members and study sponsors are required to keep your identity confidential. Your file will be kept in a locked file cabinet and will be accessible only to the researcher. To ensure that the research is conducted in accordance with regulations, members of the government administration or ethics review committee will have access to your personal information at the research unit, as required. When the results of this study are published, no personal information about you will be disclosed.

We will contact you promptly about any meaningful new developments or new medical information related to your health during the study, such as suggesting that you undergo tests to determine such new information. I will also keep you informed of any new information that may affect your choice to continue in the study.

**11. Subject rights:**

Participation in the study is entirely voluntary. You may refuse to participate in the study, or withdraw from the study at any time during the study, and your data will not be included in the study results. None of this will affect your relationship with your physician. Any medical treatment or rights you may have will not be affected by this.

The study physician may terminate your participation in the study if you require other treatment, if you fail to comply with the study plan, if you have a study-related injury, or for any other reason.

**Informed Consent Form Signature Page**

I have read this informed consent form and have discussed and asked questions about this study with my doctor. I have been given a detailed explanation of the purpose of the study, the study process, the possible risks and benefits, and all my questions have been answered, and I understand that participation in this study is voluntary.

I acknowledge that I have had sufficient time to consider this, including the possible risks of participating in the study. I am aware that I can consult with my physician at any time for further information, that I can withdraw from the study at any time without discrimination or reprisal, that my medical treatment and benefits will not be affected by withdrawal from the study, and that it would be in my interest and that of the study as a whole to inform my physician of any changes in my condition and to complete appropriate physical and physical examinations if I withdraw from the study, particularly for treatment reasons. If I need to take any other treatment as a result of a change in my condition, I will seek prior advice from my physician or tell him/her truthfully afterwards.

I am voluntarily participating in this study. I give my consent to the investigator, sponsor, health administration supervisory authority/drug and food regulatory authority, and ethics committee to access my study data.

I will be given a signed and dated copy of the informed consent.

Subject’s name: _____________. Proxy’s name: _____________.

Subject’s signature: __________. Proxy’s signature: _____________.

Date: _____________. Date: _____________.

(Note: Signature of witness required if subject is illiterate and signature of agent required if subject is incapacitated)

I have accurately informed the subject of this document and he/she has accurately read this informed consent form and certify that the subject has had the opportunity to ask questions. I certify that he/she has given voluntary consent.

Name of Investigator: ________________________

Investigator's signature: _________________________

Date: _________________________
